# Editorial: Omics-originated exploration of pathogenic patterns and molecular mechanisms in human and animal fungal pathogens

**DOI:** 10.3389/fmicb.2023.1243709

**Published:** 2023-07-11

**Authors:** Chuan Xu, David Kadosh, Donglei Sun, Guohong Zeng, Yi-Zhou Gao

**Affiliations:** ^1^Key Laboratory for the Genetics of Developmental and Neuropsychiatric Disorders, Ministry of Education, Bio-X Institutes, Shanghai Jiao Tong University, Shanghai, China; ^2^Department of Microbiology, Immunology and Molecular Genetics, University of Texas Health Science Center at San Antonio, San Antonio, TX, United States; ^3^School of Life Sciences and Biotechnology, Shanghai Jiao Tong University, Shanghai, China; ^4^Zhejiang Province Key Laboratory of Plant Secondary Metabolism and Regulation, College of Life Science and Medicine, Zhejiang Sci-Tech University, Hangzhou, China; ^5^Center for Microbes, Development and Health, Key Laboratory of Molecular Virology and Immunology, Chinese Academy of Sciences, Shanghai, China

**Keywords:** omics, genome-wide patterns, pathogenic factors, molecular regulatory mechanisms, disease, biocontrol, human and animal fungal pathogens

While fungal pathogens can cause serious diseases in humans, they can also infect animals to prevent the transmission of threatening human diseases. Regardless of the positive or negative effects on human health, we need to gain a deeper understanding of their biology. High-throughput omics technologies have largely accelerated life sciences, but most of the advances and applications have been limited to model species and they have not significantly promoted fungal pathogenesis research. It is therefore of interest to encourage the application of omics technologies for research on human and animal fungal pathogens. Within this context, we launched our Research Topic and invited researchers to explore fungal pathogenic patterns and molecular mechanisms through high-throughput omics technologies.

This Research Topic has collected six recent studies originating from multiple omics technologies, including genomics, transcriptomics, metabolomics, metagenomics, and the gut microbiome. The pathogenic fungi involved in this topic include the animal pathogen *Metschnikowia bicuspidate*, human pathogens *Candida albicans, Cryptococcus neoformans*, and many gut fungi in rats or humans. The papers provide valuable insights into the application of omics technologies to explore human and animal fungal pathogens and accelerate our understanding of fungal pathogenesis.

*M. bicuspidata* is a globally distributed pathogenic yeast, which has caused a serious reduction in production and marked economic loss for the aquaculture industry. Jang, Bao, Xing, Cao et al. isolated a new strain of this species from the Chinese mitten crab *Eriocheir sinensis*, and sequenced its genome. They carried out a high-quality genome assembly and obtained a 16.13 Mb draft genome. They predicted 5,567 putative genes for the genome, among which 331 were unique to the strain and 30 were putatively related to pathogenicity. Since many fungal pathogenesis studies are based on genome sequences, this study will lead to a significantly improved understanding of *M. bicuspidate* pathogenesis as well as therapeutic interventions for treatment of *M. bicuspidata* infections.

Another study, also from Jang, Bao, Xing, Li et al. instead focused on the infection of *M. bicuspidate* in its host. The authors investigated changes of *E. sinensis* metabolism and gut microbiota following infection by *M. bicuspidata*. They found that fungal infection leads to significant changes in the levels of 420 compounds that were enriched in 58 metabolic pathways. They also found that upon *M. bicuspidata* infection, anaerobes were increased and ascomycetes were decreased in the gut of *E. sinensis*. This study provides an important reference for the pathogenesis of crab fungal diseases, as well as potential ideas for the prevention and treatment of these diseases in the future.

Li H. et al. compared gut microbiota between patients with cryptococcal meningitis (CM) and healthy controls and determined the effects of antifungal drugs. The authors reported altered bacterial and fungal microflora in CM patients. They also found the composition of gut microbiota is associated with clinical symptoms. For example, auditory symptoms are positively correlated with *Enterococcus lactis* and *Saccharomyces cerevisiae*. Unexpectedly, treatment with antifungal drugs increased the richness of bacteria and fungi in most individuals, leaving questions about why these drugs did not reduce fungal diversity for future investigation. This study provides novel information that will serve as the basis for future larger population-based and mechanistic studies.

Another microbiome study by Li W. et al. investigated whether prednisone changed the composition of gut fungi and the interactions between the gut mycobiome and bacteriome/fecal metabolome in rats. The authors found no changes in the richness of the gut mycobiome in rats following prednisone treatment, but the diversity increased significantly. Specifically, the relative abundance of *Aspergillus glabripes* increased substantially, while *Triangularia mangenotii* and *Ciliophora* sp. decreased. They also found altered gut fungi-bacteria interactions in rats following prednisone treatment. In addition, they found some fungal genuses, such as *Triangularia* and *Ciliophora*, were correlated with many metabolites. In conclusion, this study demonstrates that prednisone treatment causes fungal microbiota dysbiosis and might alter the ecological interaction between the gut mycobiome and bacteriome in rats.

Yang et al. explored the correlation between *C. albicans* and the oral microbiome. Based on metagenomic sequencing, they compared the supragingival microbiota between 23 healthy patients and 31 patients with white spot lesions (WSL) undergoing fixed orthodontic treatment, and found that *C. albicans* is enriched in orthodontic derived white spot lesions and plays potential roles in shaping the focal supragingival bacteriome. Specifically, the WSL group is composed of fewer *Neisseria* and *Cardiobacterium* species but more *Leptotrichia* species compared to that of the healthy group. In the WSL group, 45% of patients were *C. albicans* carriers while *C. albicans* was not detected in the healthy group. This study creatively proposed a potential relationship between WSL and *Candida*, which has important scientific significance in the complex ecological relationship between bacteria, fungi, and hosts.

Li Z. et al. examined the antifungal effects of a plant-derived chemical, allicin. Using *C. neoformans* as the testing subject, the authors evaluated antifungal effects by comparing allicin to the commercially available antifungal drugs fluconazole and amphotericin B. They found that allicin has comparable antifungal activity to that of fluconazole and is even more effective against amphotericin B resistant strains. Using transcriptomics analysis, combined with electron microscopy and live cell imaging, the authors found that the mechanism of allicin antifungal effect occurs through damage and permeabilization of fungal cell membrane. This study focused on antifungal effects and mechanisms of drug action, which will contribute to the development of more effective therapies for treatment of *C. neoformans* infections.

Taken together, the studies included in this Research Topic highlight how using high-throughput omics technologies for fungal pathogens can provide a more expansive view of pathogenic patterns and molecular mechanisms. These studies also showcase the microbial ecological effects of fungal pathogens in humans and animals. The application of many other omics technologies widely used in model species to human and animal fungal pathogens would further strengthen this collection. However, we are confident that all the selected studies in our Research Topic bring important and enduring contributions to our understanding of fungal pathogens, which can also be valuable to fungal biology.

## Author contributions

CX, DK, DS, GZ, and Y-ZG contributed to the writing and editing of the manuscript. All authors contributed to the article and approved the submitted version.

